# Analysis of Cryogenic Impact Properties for a Glass-Fiber-Reinforced Dicyclopentadiene with a Different Amount of Decelerator Solution

**DOI:** 10.3390/ma12193246

**Published:** 2019-10-04

**Authors:** Ji Ho Jeon, Woo Il Lee, Jong Min Choi, Sung Woong Choi

**Affiliations:** 1 Department of Mechanical and Aerospace Engineering, Seoul National University, Seoul 08826, Korea; jeonjiho1114@gmail.com (J.H.J.); wilee@snu.ac.kr (W.I.L.); 2 Department of Naval Architecture and Ocean Engineering, Pusan National University, Busan 46241, Korea; mingun@pusan.ac.kr; 3 Department of Mechanical System Engineering, Gyeongsang National University, Tongyeong-si 53064, Korea

**Keywords:** charpy impact test, decelerators, glass-fiber-reinforced polydicyclopentadiene (p-DCPD)

## Abstract

Composites using dicyclopentadiene (DCPD) as a matrix have gained significant popularity owing to their excellent impact and chemical corrosion resistance. In the present study, experiments addressing the impact behavior of glass-fiber-reinforced DCPD were conducted to quantitatively evaluate its impact properties. The glass-fiber-reinforced polydicyclopentadiene composite utilized in impact tests was manufactured using structural reaction injection molding (S-RIM) because of its fast curing characteristics and low viscosity. The impact properties of the glass-fiber-reinforced DCPD (GF/DCPD) were quantitatively evaluated by varying its fiber content and decelerator solution. The impact properties of neat DCPD and GF/DCPD composites were examined with different amounts of decelerator solution under various temperatures from room temperature to cryogenic temperature to observe the ductile-to-brittle transition temperature (DBTT). With an increase in the fiber weight fraction of the GF/DCPD composite, the effect of the DBTT significantly decreased. However, the decreasing rate retarded as the weight fraction of the GF increased. The decreased DBTT with the addition of GF in the GF/DCPD can be attributed to the differences in the thermal expansion ratio and the interfacial force between neat DCPD and the fiber. A fractograph analysis demonstrates that the effect of the brittle (smooth) surface resulted in a lower impact absorbed energy when the temperature decreased, along with the increased amount of the decelerator.

## 1. Introduction

Liquid composite molding (LCM) processes have been significantly used over the past few decades for the composite manufacturing required for complex structures. Composite materials are used for various applications, such as sporting goods and automotive products, as well as in the military and aerospace fields, owing to their ease in processing, low weight, and cost-effectiveness [1,2]. In the LCM processes, traditional manufacturing techniques are used for manufacturing conventional fiber-reinforced polymer composites and thermoplastics, including resin transfer molding (RTM), vacuum infusion, compression molding, direct extrusion, compounding, and injection molding [3].

Generally, epoxy resins are most well known as a thermosetting resin used in the LCM process. However, owing to their lack of thermal stability, flammability, and long curing time, a significant amount of effort has been undertaken to determine adoptable resins for the LCM process.

Because industries are determined to reduce manufacturing cost and time, composites using dicyclopentadiene (DCPD) as a matrix have gained significant popularity owing to its excellent impact and chemical corrosion resistance [4]. DCPD has a colorless liquid with a low viscosity which a monomer commercially derived from petrochemicals. Poly-dicyclopentadiene (poly-DCPD) is formed by ring-opening metathesis polymerization (ROMP), activated by a catalyst. Titanium-, molybdenum-, chromium-, and ruthenium-based catalysts are generally used; however, in this study, ruthenium-based catalyst was used because polymerization can proceed under atmospheric conditions.

Because of the advancement in composite fabrication methods and materials underpinning their improved properties, industrial applications, such as those involving fuel tanks, pipes, support elements, vessels, and electrical insulation, have made good use of these materials [5].

Among the variety of composite materials, DCPD resins are significantly favored owing to their excellent physical and mechanical properties as well as their compatibility for cryogenic application [6]. Composite materials used under the cryogenic environment must be structurally reliable to maintain their performance during their entire service. Examining the impact property is one of crucial factors for determining adaptability in a cryogenic environment.

Various studies have been conducted on the material performance of new types of resin, particularly with regard to impact properties to estimate toughness. Laura et al. [7] examined the impact strength and tensile properties of Nylon 6 and maleated ethylene–propylene rubber (EPR-g-MA) reinforced with glass fiber as a function of glass fiber and EPR-g-MA content. Tjong et al. [8] investigated the impact fracture resistance of injection-molded PA6/SEBS-g-MA blend and its hybrid composites. Using the Charpy impact test, they demonstrated that hybrids exhibit a much higher notched impact strength than the PA6, 6 polymer under various test velocities of 1–5 m s^−1^. Daiyan et al. [9] studied the characteristics of the low-velocity, low-energy impact response of mineral and elastomer-modified polypropylene from the effects of plate thickness, impact velocity, and temperature [9]. They also examined the effect of molding conditions and other effects, such as striker geometry, clamping, surface texture, weld line, and paint effects [10]. Yoo et al. [11] carried out the Izod impact test to measure the impact property of the Nylon 6 composites, containing both organoclay and glass fibers as fillers, which were prepared by melt processing. They demonstrated that the impact strength increased with glass fiber content but decreased with clay content. Consequently, the impact property was frequently measured to estimate toughness. However, for most impact tests, the composite materials were conducted under atmospheric conditions without influence due to cryogenic temperature effects.

The effect of cryogenic temperature on composite materials has been partially demonstrated in a limited number of studies. Fu et al. [12] studied the tensile and impact properties of matrices and nanocomposites under a temperature of −196 °C. The epoxy-blended matrix was prepared by incorporating polyurethane-epoxy into diglycidyl ether of bisphenol-F (DGEBF)-type epoxy, and SiO_2_/epoxy nanocomposites were prepared via the sol-gel process, incorporating DGEBF-type epoxy and tetraethylorthosilicate (TEOS). Yang et al. [13] investigated the cryogenic mechanical behavior of the epoxy resins in terms of tensile properties and Charpy impact strength at the low temperature of −196 °C. The results were then compared to their corresponding behavior at 25 °C. For the same composition, they demonstrated that the tensile strength and the Young’s modulus at −196 °C were higher than those at 25 °C. However, the elongation at break and impact strength exhibited opposite behavior. Yang et al. [14] found improvements in mechanical properties at low temperatures for epoxy resins such as diglycidyl ether of bisphenol A (DGEBA). They concluded that the tensile strength, failure strain (ductility), and impact strength at −196 °C were simultaneously improved by adding the proper amount of a hydroxyl functionalized hyper-brached polymer (H30). In fact, the identification of the temperature-dependent ductile–to-brittle transition temperature (DBTT) is essential for determining the operating temperature range of the material. Nevertheless, there have been very few studies on the DBTT of composite materials. Kaiser et al. [15] conducted an experimental study to determine the ductile-to-brittle transition of various PLA-based bio-composites—PLA, PLA-20KF (Kenaf fiber), PLA-20KF-5Clay, and PLA-5Clay—through impact tests at temperatures ranging from −5 °C to 28 °C. Flexman [16] examined the ductile-brittle transitions of nylon-66 compositions by Izod and falling weight tests. However, owing to the limited number of studies on DBTT for the new type of resin-DCPD/composite materials, the reliability of choosing DCPD remains unclear. Furthermore, additional studies examining the effect of the catalysts resulting from the addition of a decelerator to the DCPD should be conducted to ensure material reliability under various operating conditions. Our previous study on DCPD with different decelerator solutions was carried out to obtain curing kinetics with decelerator effects [17].

In this study, experiments for the impact behavior of glass-fiber-reinforced DCPD was investigated to quantitatively evaluate the impact properties. To investigate the effect of the fiber content, the impact properties of the glass-fiber-reinforced DCPD were evaluated with different weight fractions of glass fiber and were compared with neat DCPD. Furthermore, to ensure various environmental conditions, the impact properties of DCPD were investigated with different amounts of decelerator solution under various temperatures, particularly at cryogenic temperatures to find out cryogenic reliability. Finally, the fracture surfaces were analyzed with fractograph analysis to investigate the effects of the fracture toughness of DCPD.

## 2. Experimental

### 2.1. Materials

**Figure 1:**
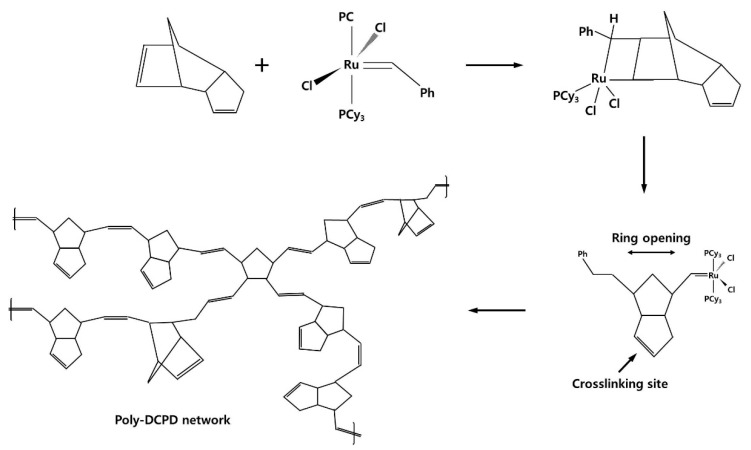
The polymerization of dicyclopentadiene (DCPD) with Grubbs catalyst [17].

The monomer and catalyst used in this study was Dicyclopentadiene monomer (endo-DCPD, 95%, stabilized with 100–200 ppm 4-tert-butylcatechol, Sigma-Aldrich, St. Louis, USA) and Ru-based Grubbs 2nd catalyst (2nd generation, Sigma-Aldrich), respectively. The chemical formulae [18] of the reactants are represented in Figure 1 [19]. In our previous study [19], Ru-based Grubbs 2nd catalyst was found to be an optimized catalyst. To accelerate the dissolution of the catalyst, 0.2 mass% was dissolved in a toluene solvent. The decelerator retarded the curing of DCPD with an amount ranging between 1–2 mass. % (Triphenylphosphine, 95%, Sigma-Aldrich). This was found to be a suitable range for the amount of DCPD in our previous study [17]. With different amounts of decelerator, endo-DCPD monomer mixed with the catalyst. The monomer and catalyst were blended together by stirring mechanically at 25 °C for 10 s in a vial [19].

### 2.2. Test Specimen

**Figure 2:**
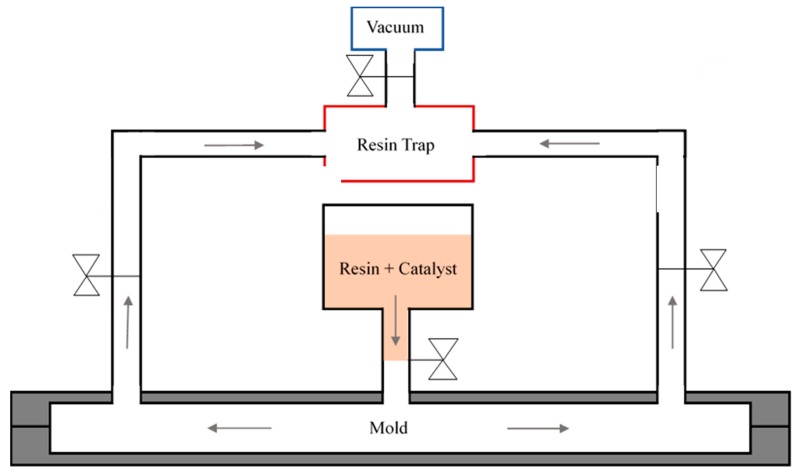
The schematic illustration of the experimental apparatus for the structural reaction injection molding (S-RIM).

The test specimen prepared for the impact tests was manufactured using the structural reaction injection molding (S-RIM) manufacturing process because DCPD is a fast-curing resin with low viscosity. The schematic illustration of the S-RIM apparatus is shown in Figure 2. The vacuum pump was connected to the resin vessel to remove any unwanted void, and vacuum is applied within the resin trap such that excess resin from the mold can be stored.

In the condition of air-free nitrogen atmosphere with ventilation system, the DCPD resin and catalyst were mixed in the mixing head thoroughly and injected into the mold. The DCPD in the mold was cured by increasing the mold temperature up to 100 °C with a curing time of 30 min. The surface treatment was used to facilitate adhesion between the fiber and the DCPD resin. After an adhesion treatment by the deposition of a liquid phase of Trichloro (1H, 1H, 2H, 2H - Perfluorooctyl) Silane (97%, Sigma-Aldrich, St. Louis, USA) for 10 min, the upper mold was rinsed with ethanol and acetone [19]. To examine the effect of the glass fiber content, samples of DCPD were prepared including the neat version and the specimens with different weight fractions of glass fiber ranging between 10–30 wt.%. The glass fiber was a preformed chopped glass fiber with a density of 450 g/cm^2^.

### 2.3. Charpy Impact Test

The Charpy impact test is a standardized high-strain-rate impact test to obtain amount of energy absorbed during the material fracture at test temperatures. The absorbed energy in a Charpy impact test can be calculated as follows:

(1) E=MRg(cosβ−cosα)

where *E*, *M*, *g*, *β*, and *α* is the energy absorbed in a Charpy impact test, the hammer’s mass, the gravitational acceleration, the angle at the end of the swing, and the angle of fall, respectively. Chapy impact tests were conducted using a pendulum-type impact apparatus (TM-CIMC, Max 500J, Testmate. Co., Gimhae-si, Korea) and test samples were fabricated in accordance with ISO 179 [20]. This is a standard method of obtaining the absorbed energy from the pendulum impact on plastics. The dimension of the specimens investigated in this study was characterized by a 10 mm × 4 mm section, with a length of 80 mm. The notches in the specimens were formed with a central 45° V-notch, with a depth of 2.54 mm and a root radius of 0.25 mm in accordance with ISO 179 [20].

### 2.4. Experimental Conditions

To investigate the temperature effect, temperature conditions for the experimental impact test was set in the range between the RT (25 °C) and CT (−160 °C). Experiments using the impact test were carried out for five different temperatures (25, −20, −60, −110, and −160 °C), to generate DBTT trends. To provide environments for various temperatures, temperature chamber was used where liquefied nitrogen was injected into a chamber and testing temperature are automatically controlled with solenoid valve. Except for the specimen tested at the room temperature, all the specimens were pre-cooled at the test temperature for approximately 60 min to maintain their thermal equilibrium. The tests in each case were performed at least five times to ascertain the repeatability of the test results. The fracture surfaces were examined using a microscope (3D profiler, KH-8700, Hirox Korea Co., Ltd., Anyang-si, Korea).

## 3. Results and Discussion

### 3.1. Observation of Glass Temperature

**Figure 3:**
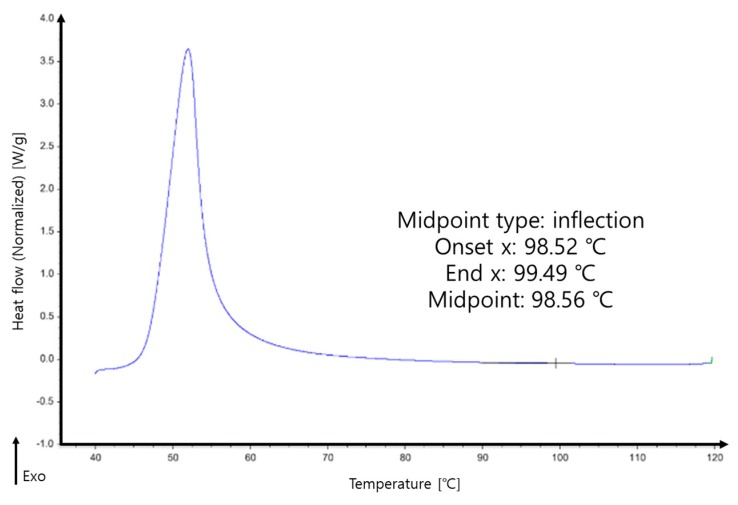
Scanned glass transition temperature of the cured materials of DCPD.

Thermal properties of the neat DCPD and DCPD with decelerators were measured using differential scanning calorimetry (DSC) because DSC is the most common method to determine glass transition which detects the change in heat capacity. The temperature was scanned to obtain the glass transition temperature of the cured materials in terms of the change in heat capacity, defined as the glass transition temperature (Tg), as shown in Figure 3. It was defined by the intersection point of the two tangential lines drawn along the discontinuous specific heat with temperature profiles.

The variation in Tg with respect to decelerators is given in Table 1. The Tg of the neat cured DCPD was 105.49 °C, which was much smaller than that of the epoxy resin of 131.1 °C [21]. Hence, the change in the specific heat of neat DCPD is much smaller than that of the cured neat epoxy resin [21].

As summarized in Table 1, the Tg of DCPD decreased by 11% with the addition of 2 wt.% decelerator. The decelerator effect of endo-DCPD was analyzed in our earlier studies [17], where we found that the curing reaction of DCPD was hindered by the decelerator. As the contents of the decelerator increased, the crosslinking density decreased, leading to a decreased curing reaction. Accordingly, the Tg of DCPD decreased with the decelerator.

### 3.2. DBTT for the Neat DCPD and DCPD with Decelerators

**Figure 4:**
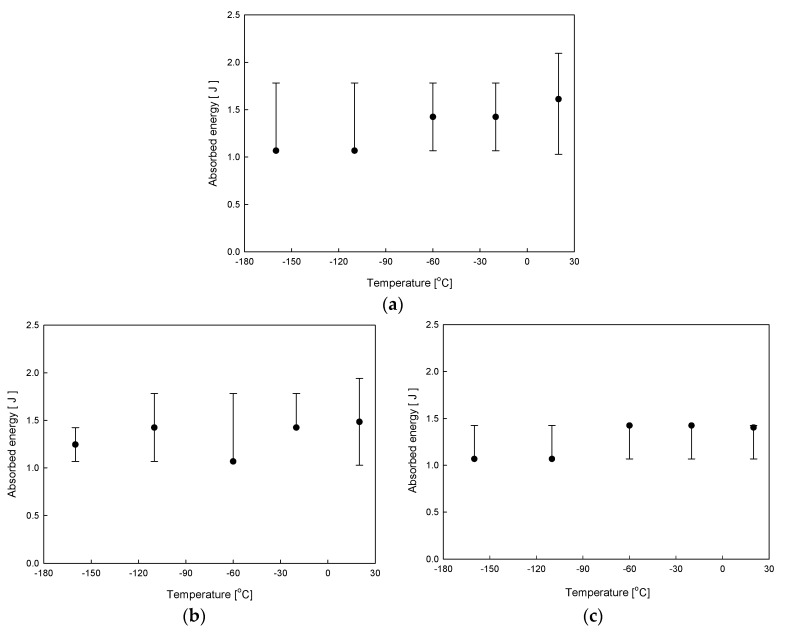
Impact absorbed energy for DCPD with decelerators of (**a**) neat DCPD, (**b**) 1 wt.% and (**c**) 2 wt.%.

The most common method to measure DBTT is by implementing the Charpy impact energy observation. During the impact test, the energy absorbed from the fracture of the specimen was calculated by using Equation (1). Likewise, the Charpy impact strength can be calculated by dividing the absorbed energy by the cross-sectional area to determine the DBTT [15]. Experimental temperatures set for specimens were in the range between the RT (room temperature) and CT (−160 °C). The temperature dependence of the impact absorbed energy for neat DCPD from RT to CT is shown in Figure 4. The standard deviation is represented as the error bars. As can be seen in Figure 4, the impact absorbed energy decreased from RT to CT by 19%.

Figure 4b,c shows the temperature dependence of the impact absorbed energy for the DCPD with decelerators of 1 and 2 wt.% from RT to CT. With the decelerator, the impact behavior of 1 wt.% and 2 wt.% decreased by 16% and 9%, respectively, under CT compared to RT. A rapid decrease in the impact absorbed energy was observed for neat DCPD, and the decreasing rate was retarded with an addition of decelerators, exhibiting a slight reduction of the impact energy for the DCPD with 2 wt.% decelerator. With the decreased temperature, the chain in the polymer is rigidly bounded. In these circumstances, the absorbed impact energy was demonstrated to decrease [15].

### 3.3. DBTT for the GF/DCPD Composite with Different Weight Fractions

**Figure 5:**
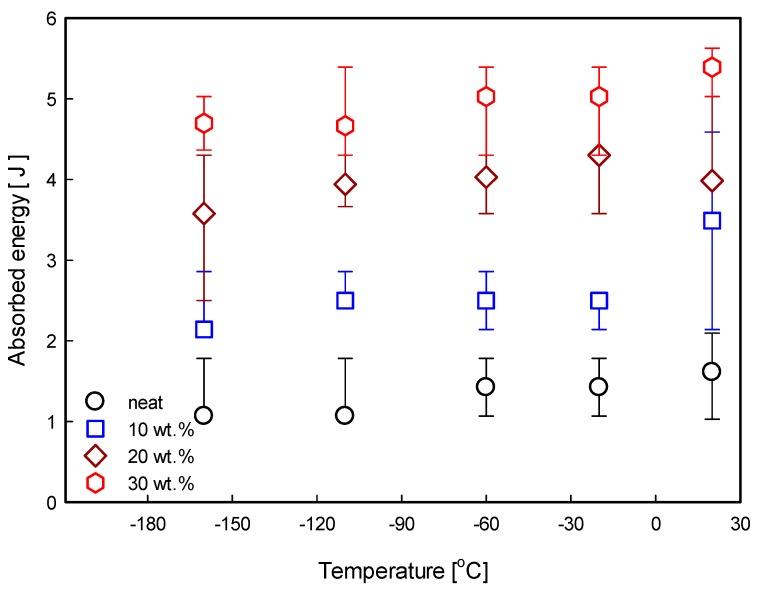
DBTT for glass-fiber-reinforced DCPD (GF/DCPD) composites with different weight fractions.

The effect of the fiber weight fraction for the GF/DCPD composite on impact property behavior has been observed. Figure 5 shows the DBTT for the GF/DCPD composites with different weight fractions.

The most crucial observation was that the DBTT of the GF/DCPD composite significantly reduced the impact strength with the GF. Furthermore, the decreasing rate was retarded as the weight fraction of the GF increased. The impact absorbed energy increased with an increase in the GF, as expected. However, the absorbed energy decreased gradually as the experimental temperature decreased, indicating the DBTT. For 10 wt.% GF, the impact strength dramatically decreased below −20 °C. For 30 wt.% GF, the decreasing interval was demonstrated to be below −60 °C. The temperature interval at which the impact strength suddenly decreased was found to fall with increasing GF weight fractions, exhibiting different DBTT behavior. This was mainly due to two critical reasons. The first reason pertains to the difference in the thermal expansion ratio between neat DCPD and the fiber. Generally, thermal contraction at CT (cryogenic temperature, −160 °C) was found for both resin and the fiber. However, since the resin has a higher thermal contraction over the fiber, the occurrence of DBTT for lower fiber content specimens occurs at a relatively higher temperature in comparison to those with higher fiber content. Therefore, for the lower fiber content specimens, the resin thermal contraction was predominant over the fiber thermal contraction. However, as the fiber weight increases, the DBTT reduces to a lower temperature, indicating that the effect of fiber was more influential than that of the resin. Furthermore, as the proportion of the thermal contraction from the fiber increases owing to an increase in the fiber content, the reduction rate of the impact property over the decreasing temperature declined.

Another plausible reason is the difference in the interfacial force between the DCPD resin and the fiber. With decreasing temperature, the interfacial force between the DCPD resin and the fiber is increased. By relating back to the thermal contraction of fibers and the resin, greater interfacial bond was achieved as the temperature was lowered because the higher thermal contraction of the resin resulted a tightening of the fiber [22]. This means that the large extent of the contraction in the DCPD resin tightened the fibers, leading to an increase in the resistance during the fiber pull-out [22]. The resistance during the pulling of fibers from friction resulted in a lower dissipation of impact absorbed energy. This implies that the temperature at which the DBTT occur is delayed for specimens with higher fiber contents owing to the decreasing effect of the resin thermal contraction.

### 3.4. Decelerator Effect with Different Weight Fractions

**Figure 6:**
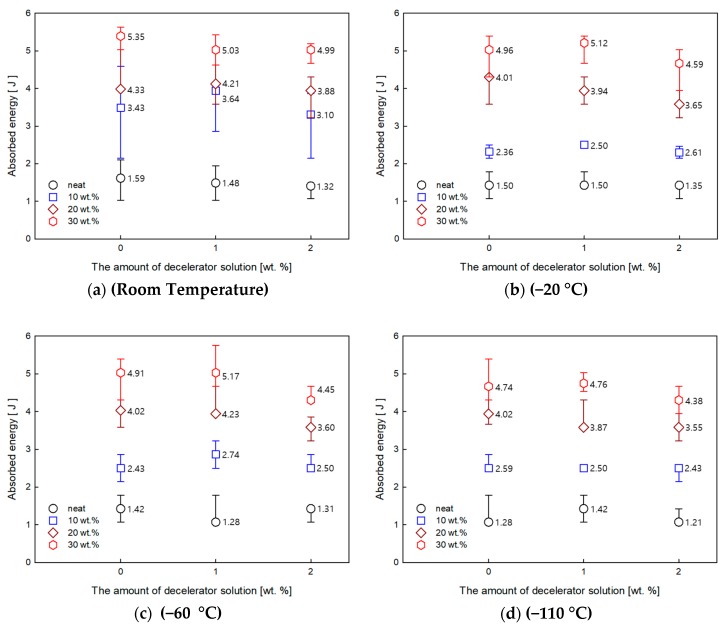

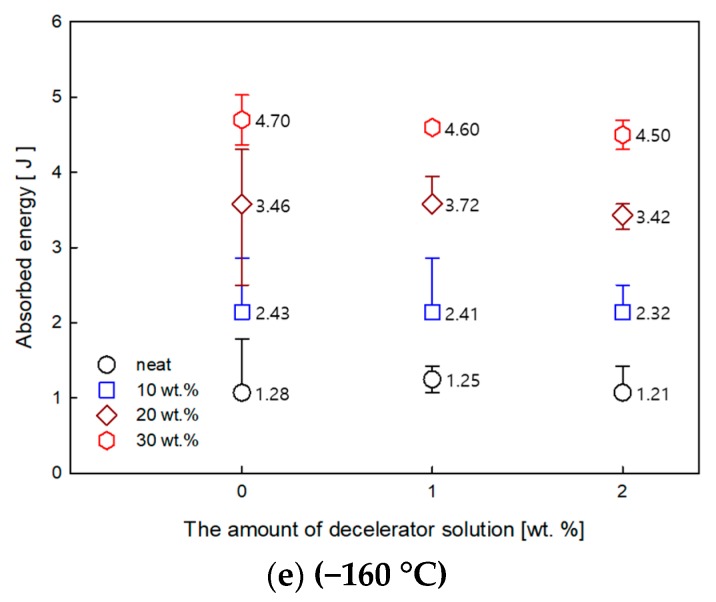
Impact strength of neat and GF/DCPD composites as a function of temperature with different amounts of decelerator. (**a**) Room Temperature, (**b**) −20 °C, (**c**) −60 °C, (**d**) −110 °C, (**e**) −160 °C

An observation of the impact property was undertaken to evaluate the decelerator effect on the DBTT of GF/DCPD composite. Figure 6 show the impact strength of the neat and GF/DCPD composites as a function of temperature with different amounts of decelerator. As the temperature decreased with the amount of decelerator increasing, the impact absorbed energy reduction rate decreased. Under the room temperature condition, the impact energy of the neat DCPD reduced by 17% as the amount of decelerator increased to 2 wt.%. Meanwhile, 7% reduction was observed for the glass fiber samples with a 30% weight fraction. However, under the cryogenic temperature condition, the impact energy of the neat DCPD and the sample with 30% glass fiber content reduced by 6 and 4%, respectively. At room temperature, it was clear that the reduction of the impact strength was due to an addition of decelerator. Conversely, at cryogenic temperature, the decreasing rate of the impact property was reduced with an addition of decelerator. This is owing to the different thermal property of DCPD: Tg with different amounts of decelerator. The decrease in Tg was attributed by a loss in the mobility of the crosslinked chain, resulting in a retarded curing reaction. With the lower value of Tg from the samples with 2 wt.% of the decelerator, a susceptibility to fracture was demonstrated, even at cryogenic temperature. Hence, the reduction rate was clearly observed at cryogenic temperature.

Moreover, with a high weight fraction of GF, the effect of the decelerator was reduced. As can be seen in the cases for 10 wt.% of GF or above, the effect from the fiber was predominant over the decelerator effect.

### 3.5. Fractograph Analysis

**Figure 7:**
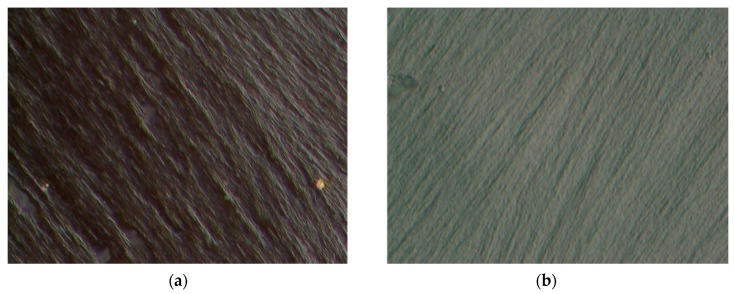
The fracture surfaces of neat DCPD ((**a**) room temperature, (**b**) cryogenic temperature, up to 2000× magnification).

**Figure 8:**
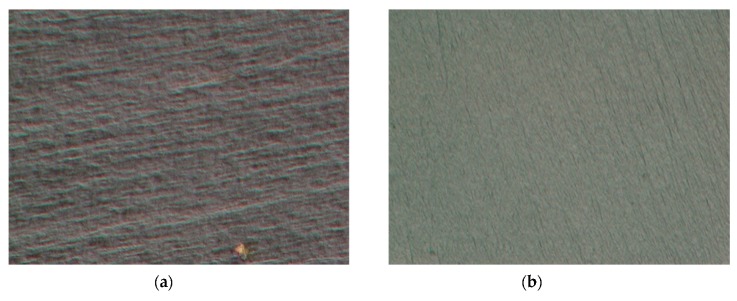
The fracture surfaces of neat DCPD with decelerator under CT (−160 °C) condition. ((**a**) 1 wt.% decelerator, (**b**) 2 wt.% decelerator, up to 2000× magnification).

To evaluate the fracture behavior of neat DCPD followed by the Charpy impact test, microscopic images of the fracture surface were observed. As can be seen in Figure 7, a variation in the fracture surface was observed for different temperatures. At CT (−160 °C), specimens of the neat DCPD demonstrated a more brittle fracture characteristics compared to those at RT. The microscopic images of the fracture surface of neat DCPD with the addition of decelerator is shown in Figure 8. As can be seen in the Figure 8, a relatively smooth surface was observed for the specimen with the addition of a decelerator. It was evident that adding a decelerator to the resin increases brittleness upon fracture. The presence of the smooth surface was characterized by a less shear deformation, and this is the reason for the lower impact absorbed energy when 2 wt.% of the decelerator was added to DCPD.

**Figure 9:**
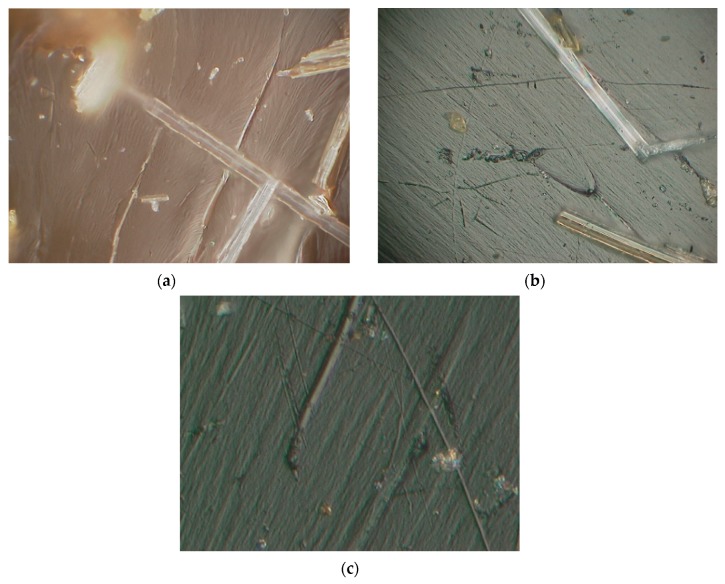
The fracture surfaces of 10 wt.% GF/p-DCPD composite ((**a**) Room temperature, (**b**) CT (cryogenic temperature, −160 °C), (**c**) CT with 2 wt.% decelerator, up to 2000× magnification).

**Figure 10:**
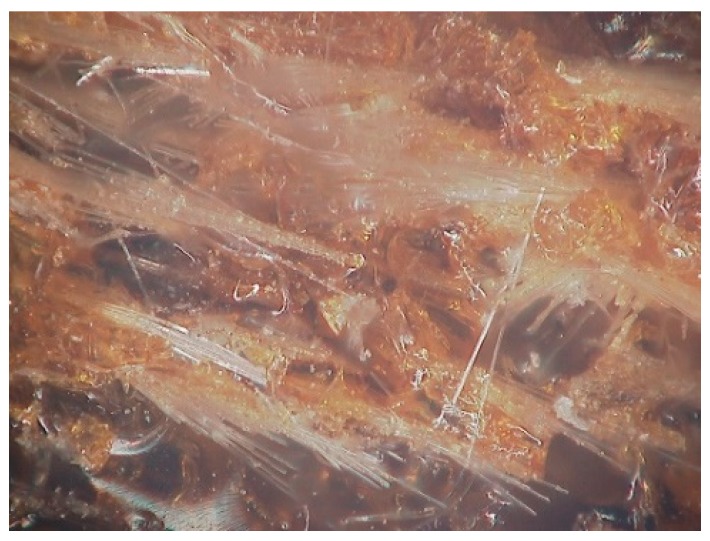
The fracture surface of 30 wt.% GF/p-DCPD composite.

The microscopic images of the fracture surfaces of the GF/p-DCPD composite after the Charpy impact tests are shown in Figure 9. For the 10 wt.% GF/p-DCPD composite, a more brittle fracture was observed at CT (−160 °C) compared to that at room temperature. Apparent brittle fracture characteristics were also demonstrated with the samples of 2 wt.% decelerator at cryogenic temperature. For the 30 wt.% GF/p-DCPD composite, however, the DCPD resin was not observed, as shown in Figure 10. The fractograph analysis results suggest that the effect from the brittle (smooth) surface was the reason for the lower impact absorbed energy when the temperature was decreased, along with the increased amount of the decelerator.

## 4. Concluding Remarks

In this study, the impact behavior of neat DCPD and GF/p-DCPD composites was investigated. The impact properties were quantitatively evaluated by varying the fiber content and decelerator solution. Furthermore, the impact properties of neat DCPD and GF/DCPD composites were examined with different amounts of decelerator solution under various temperatures, from RT (room temperature) to CT (cryogenic temperature) to observe the DBTT. The main conclusions of the study are as follows:

- Thermal properties of neat DCPD and those with a decelerator were measured using DSC. The Tg of DCPD decreased by 11% with the addition of 2 wt.% decelerator, owing to the reduction in the crosslinking density in the polymer chain with increasing decelerator, leading to a decreased curing reaction.
- The temperature dependence of the impact absorbed energy for neat DCPD from RT to CT was observed. The impact absorbed energy decreased from RT to CT by 19%. With the decelerator, the impact behavior of 1 wt.% and 2 wt.% decreased by 16% and 9%, respectively, under CT compared to RT.
- With the variations in the fiber weight fractions of the GF/DCPD composite, the effect of the DBTT significantly decreased for GF/DCPD composites. The decreasing rate was retarded, however, as the weight fraction of the GF increased. The absorbed energy decreased gradually as the experimental temperature decreased, indicating the DBTT.
- The decrease in DBTT with the additions of GF for the GF/DCPD can be explained with differences in the thermal expansion ratio and interfacial force between neat DCPD and the fiber.
- The effect of the weight percent of the decelerator on the DBTT of GF/DCPD composites as a function of temperature was clearly observed. The decreasing rate of the impact absorbed energy under decreasing temperature declined as the amount of the decelerator was increased, owing to the different thermal property of DCPD and Tg with different amounts of decelerator.
- The micrographs of the fracture surfaces of neat DCPD after the Charpy impact tests were observed. It demonstrated that the neat DCPD specimens had typical characteristics of brittle fracture, indicating a greater brittle fractured surface at CT than at RT. The fracture surface of neat DCPD with the addition of a decelerator provides the reason for the lower impact of absorbed energy when 2 wt.% of the decelerator was added to DCPD.
- In our future research, we evaluate the tensile properties dependent on temperature, particularly near the cryogenic temperature.

## Figures and Tables

**Table 1:** Glass transition temperature (Tg) of endo-DCPD and endo-DCPD with decelerators after curing reaction.

Decelerator Content (wt.%)	Neat	1	2
Tg	105.49	98.56	93.43
